# Clinicopathological characteristics, treatment and prognosis of head & neck small cell carcinoma: a SEER population-based study

**DOI:** 10.1186/s12885-020-07522-9

**Published:** 2020-12-07

**Authors:** Chen-xi Yu, Feiluore Yibulayin, Lei Feng, Meng Wang, Meng-meng Lu, Yuan Luo, Hui Liu, Zhi-cheng Yang, Alimujiang Wushou

**Affiliations:** 1 Department of Oral & Maxillofacial Surgery and Oral Biomedical Engineering Laboratory, Shanghai Stomatological Hospital, Fudan University, 356 Beijing East Road, Shanghai, 200001 People’s Republic of China; 2 Department of Clinical Medicine, Shanghai Medical College, Fudan University, 138 Yi xue yuan Road, Shanghai, 200001 People’s Republic of China; 3 Department of Preventive Medicine, School of Public Health, Shanghai Medical College, Fudan University, 138 Yi xue yuan Road, Shanghai, 200001 People’s Republic of China

**Keywords:** Small cell carcinomas, Head & neck, SEER analysis, Survival, Prognostic model

## Abstract

**Background:**

To investigate the clinicopathological characteristics of head and neck small cell carcinoma (H&NSmCC) and identify prognostic factors on the basis of the Surveillance, Epidemiology and End Results (SEER) database.

**Methods:**

Total of 789 primary cases from 1973 to 2016 were included. Univariate and multivariate analyses were performed to identify independent prognostic indicators. An H&NSmCC-specific nomogram was constructed and compared with the AJCC staging system by calculating the time-dependent area under the curve (AUC) of the receiver operating characteristic (ROC) curves.

**Results:**

The incidence of H&NSmCC peaked during the period of 50 to 70 years old, and the most frequent location was the salivary gland. The 5-year disease specific survival (DSS) was 27%. In the multivariate survival analysis, AJCC III + IV stage [HR = 2.5, *P* = 0.03, I + II stage as Ref], positive N stage [HR = 1.67, *P* = 0.05, negative N stage as Ref], positive M stage [HR = 4.12, *P* = 0.000, negative M stage as Ref] and without chemotherapy [HR = 0.56, *P* = 0.023, received chemotherapy as Ref] were independently associated with DSS. The H&NSmCC-specific nomogram was built based on the independent prognostic indicators. The nomogram demonstrated better predictive capacity than the AJCC staging system for 5-year DSS [(AUC: 0.75 vs 0.634; Harrell’s C-index (95% CI): 0.7(0.66–0.74) vs 0.59(0.55–0.62), *P* < 0.05].

**Conclusion:**

N stage, M stage, AJCC stage and chemotherapy were independent prognostic indicators included in the prognostic nomogram model, which can better predict the survival of H&NSmCC than the AJCC staging system.

## Background

Small cell carcinomas (SmCCs), with small round or oval cell shapes, are regarded to be identical to poorly differentiated neuroendocrine tumors, the most aggressive type of neuroendocrine cancer [1]. This type of cancer was first described as oat cell sarcoma by Barnard in 1926 [2]. Epidemiological studies have suggested that SmCCs occur most commonly in the lung [1]. Extrapulmonary SmCCs are rare, comprising only 2.5 to 5% of all SmCCs [3]. Owing to the tendency for regional or distant spread, it is crucial to rule out other sites of the primary tumor before making a prognosis of the disease [4]. Computed tomography and/or magnetic resonance imaging of selected sites and positron emission tomography scanning are recommended as appropriate examinations to assess the original localization of the tumor [1]. Patients with SmCCs lack early specific symptoms. Therefore, the cancer has already evolved to an advanced stage when diagnosed in most cases [5].

Head and neck SmCCs (H&NSmCC) is a rare malignancy that is more likely to present at an advanced stage. This type of cancer carries a worse prognosis than squamous cell carcinoma of the head and neck, the most frequent tumor in the head and neck [6]. H&NSmCC is considered to occur in multiple sites associated with the upper aerodigestive tract, of which the larynx is the most frequent site for SmCCs as a primary tumor in the head and neck [1]. Tumors arising in the salivary glands have a relatively better prognosis than SmCCs in the larynx, lung, and most other sites [1]. Limited cases make it difficult for investigators to explore standard treatments that can be strictly followed for the disease [7]. Accepted therapies include surgery, radiotherapy and chemotherapy [1]. Currently, the combination of radiation therapy and chemotherapy is preferred for the treatment of patients with H&NSmCC [8, 9]. Surgery is mainly reserved for patients who truly have early local disease or cases where locoregional lesions cannot be controlled [8, 10].

Due to the rarity of the cancer, previous studies were usually based on case reports or small case series. There is a lack of studies stemming from a large population. Thus, the clinicopathological characteristics of H&NSmCC cannot be summarized comprehensively. In the current study, we performed a retrospective analysis of patients with H&NSmCC based on the Surveillance, Epidemiology and End Results (SEER) database, one of the most representative publicly available databases in the US, with the following aims. First, we aim to describe the clinicopathological characteristics and survival of patients with H&NSmCC. Second, to estimate the indicators that influence the survival of the disease. Finally, to establish a disease-specific nomogram model to predict the prognosis of H&NSmCC patients based on the factors associated with prognosis.

## Methods

### Data collection

Data were obtained from the SEER database which collected cancer statistics covering approximately 34.6% of the US population. Cases of microscopically confirmed primary H&NSCC were extracted according to International Classification of Diseases in Oncology, third edition (ICD-O-3) as previously described [6]. All patients from 1973 to 2016 were characterized by sex, age, race, pathological grade, American Joint Committee on Cancer (AJCC) stage, marital status, follow-up time, the use of surgery, radiation and chemotherapy and outcome status. Not all cases in our study included all these data. The tumor site contained oral cavity, salivary gland, pharynx and larynx, nasal cavity, glottis and thyroid gland. The insurance status was recorded as any medicaid, insured and uninsured type. The SEER historic stage variable was used in this study to generally describe the extent of tumor invasion, containing localized (confined to the primary organ), regional (direct extension to adjacent organ/tissue or metastases to regional lymph nodes), distant (discontinuous metastases), and unspecified stages. Overall survival was defined as the interval between diagnosis and death or the last follow-up if alive.

### Statistical analysis

The chi-square test was applied to evaluate categorical variables. Survival curves of different variables were formed with the use of Kaplan-Meier estimates, and the values of these variables for prognosis were analyzed by applying the log-rank test. The screening criterion was *P* < 0.05 for variables entering multivariable analysis. The final disease-specific survival (DSS) nomogram was formulated according to the results of the multivariable analysis. The nomogram was subjected to 50% bootstrap resamples for internal validation. The discrimination performance metrics of the nomogram and AJCC 7th staging system were estimated by the concordance index (C-index) and the area under the curve (AUC) of the receiver operating characteristic (ROC) curve. The data in the study were analyzed by applying statistical packages in R (version 3.4.3), Empower R (http://www.empowerstats.com, Boston, Massachusetts), and Statistical Package for Social Sciences (SPSS, version 23.0).

## Results

### Basic clinicopathological characteristics of the study population

There were 789 H&NSmCC patients in the SEER database from 1976 to 2016 with a male to female ratio of 1.85:1. The median age of all patients was 64 years (2–96 years), and the incidence peaked during the period of 50 to 70 years old. The average follow-up time was 37 months (0–343 months). Among all patients, white people accounted for 86.69% (684/789). The most common location of the tumor site was the salivary gland (over 25%). The clinicopathological characteristics of all patients in the study are listed in Table 1. To eliminate the influence of comorbidities, a total of 507 patients were selected for DSS analysis, with a male to female ratio of 2.04:1 (Table 1). The average follow-up time for DSS was 36.7 months (0–320 months). Among all 507 patients, 65 had tumors of localized stage, 186 were classified as regional stage and 128 had distant stage.

**Table 1:** The summary of H&NSmCC patients’ clinico-pathologic characteristics

Clinicopathologic parameter	Disease specific survival				Overall survival			
	Alive	Dead	Total	***P***-value	Alive	Dead	Total	***P***-value
**Age**								
0–19	2	4	507	0.085	2	4	789	0.001
20–29	4	9			4	9		
30–39	12	9			12	14		
40–49	20	36			21	47		
50–59	35	89			38	119		
60–69	39	98			50	170		
70–79	22	71			27	158		
80+	12	45			15	99		
**Race**								
Black	12	31	506	0.773	12	51	788	0.624
White	123	311			145	539		
Others	10	19			11	30		
**Gender**								
Female	33	134	507	0.002	41	236	789	0.001
Male	113	227			128	384		
**Marital status at diagnosis**								
Single	31	57	482	0.011	32	75	747	0.001
Married	85	192			99	336		
Other status	21	96			27	178		
**Insurance status**								
Any Medicaid	13	18	224	0.172	14	30	333	0.040
Insured	81	101			99	177		
Uninsured	8	3			9	4		
**Tumor site**								
Oral cavity	10	29	501	0.000	12	49	781	0.000
Salivary gland	46	63			54	144		
Pharynx & Larynx	29	92			33	142		
Nasal cavity & accessory sinuses	34	48			39	80		
Glottis	21	91			24	140		
Thyroid gland	3	35			3	61		
**Pathological grade**								
Grade I	2	1	321	0.052	2	1	502	0.001
Grade II	3	3			4	3		
Grade III	31	75			37	128		
Grade IV	43	163			47	280		
**AJCC stage**								
I stage	13	11	234	0.000	15	21	334	0.000
II stage	16	9			18	14		
III stage	22	20			25	35		
IV stage	40	103			42	164		
**T stage**								
T1	34	51	254	0.594	42	88	374	0.207
T2	27	40			29	65		
T3	12	30			13	57		
T4	22	38			23	57		
**N stage**								
N0	49	45	254	0.003	54	82	374	0.005
N1	19	33			24	56		
N2	23	66			24	101		
N3	1	6			1	10		
NX	3	9			4	18		
**M stage**								
M0	90	102	254	0.000	100	183	374	0.000
M1	3	49			3	71		
MX	2	8			4	13		
**Surgery**								
Yes	79	138	217	–	94	261	356	0.549
No	0	0			0	1		
**Radiotherapy**								
Yes	31	135	507	0.000	34	248	789	0.000
No	115	226			135	372		
**Chemotherapy**								
Yes	35	140	507	0.001	43	270	789	0.000
No	111	221			126	350		

### Survival analysis

**Fig. 1:**
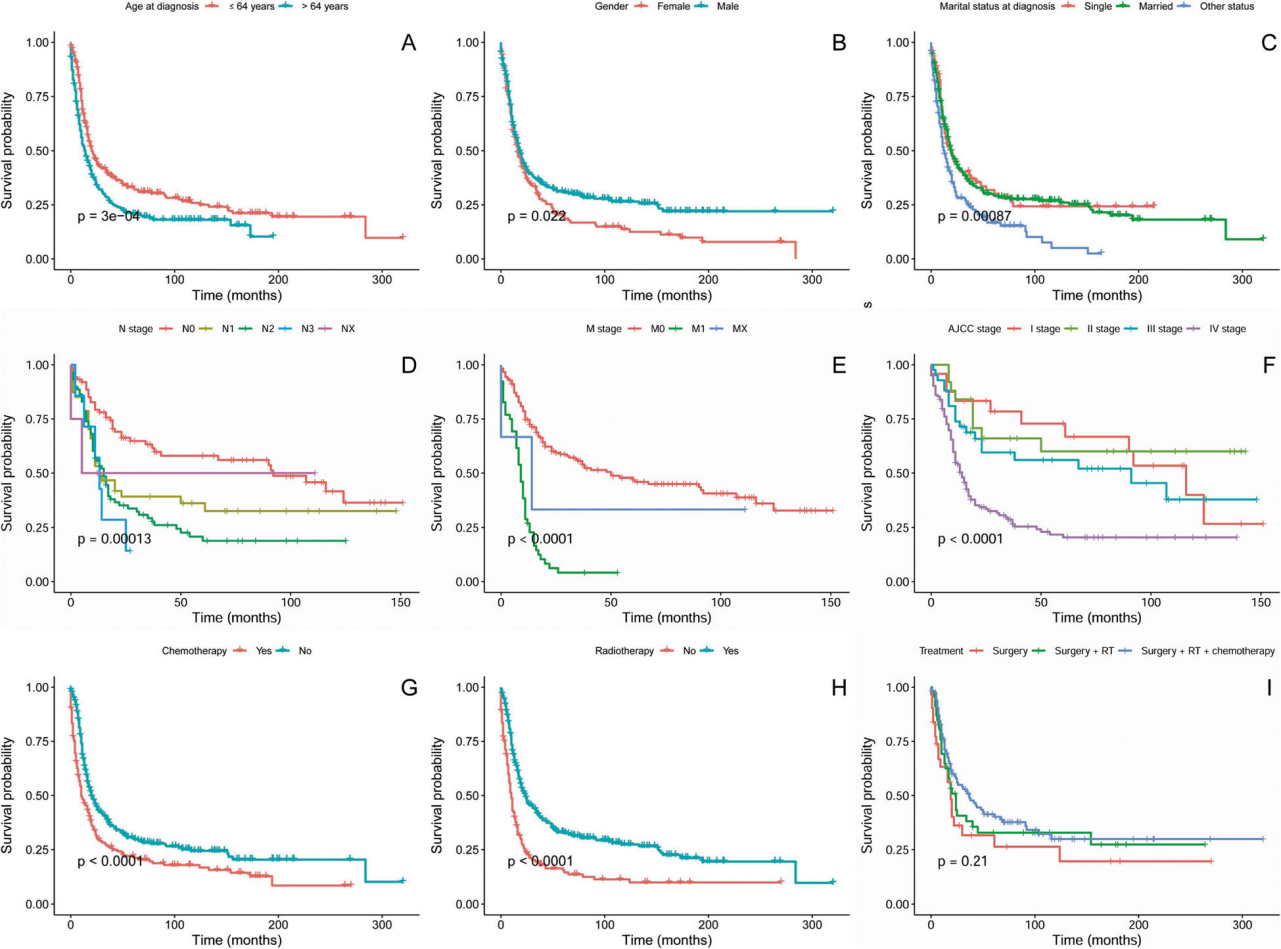
Disease specific survival curves of cases with H&NSmCC compared according to (**a**) age, (**b**) gender, (**c**) marital status at diagnosis (**d**) AJCC N stage, (**e**) AJCC M stage (**f**) AJCC stage, (**g**) chemotherapy, (**h**) radiotherapy and (**i**) treatment modalities. Log-rank test was utilized to compare curves, and significance is presented on each panel

There were significant survival differences regarding sex (*P* = 0.022), age (*P* < 0.001), marital status (*P* < 0.001), AJCC stage (*P* < 0.001), N stage (*P* < 0.001), M stage (*P* < 0.001), radiotherapy (*P* < 0.001) and chemotherapy (*P* < 0.001) for DSS (Fig. 1). The 3-year DSS, 5-year DSS and 10-year DSS were 34, 27 and 21%, respectively. The 5-year DSS was 26% for patients receiving surgery alone, 32% for those treated by both surgery and radiotherapy and 40% for others undergoing surgery, radiotherapy and chemotherapy. There was no statistically significant difference (*P* = 0.21) for the different treatment modalities.

### Cox regression analysis

**Fig. 2:**
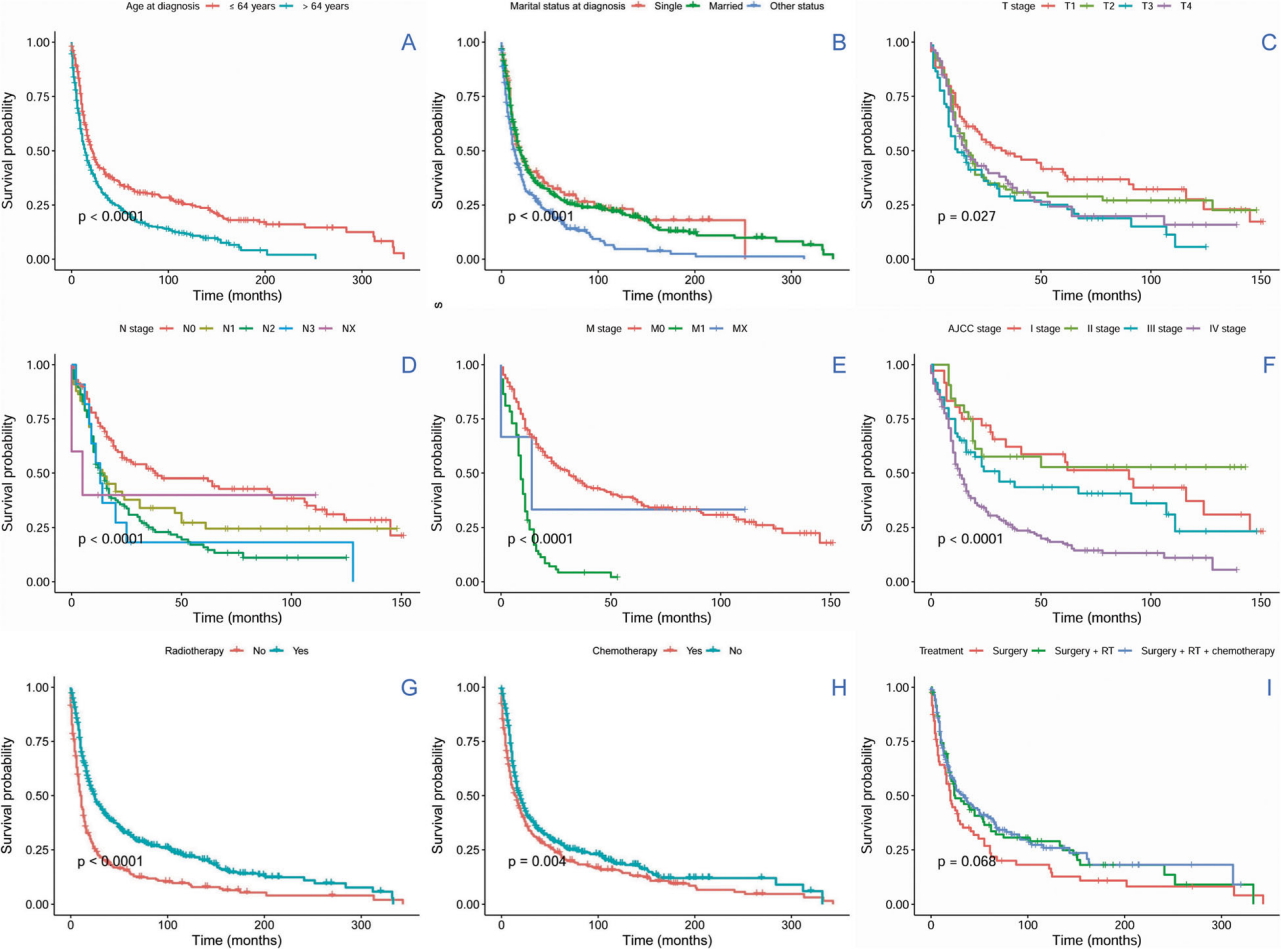
Overall survival curves of cases with H&NSmCC compared according to (**a**) age, (**b**) marital status at diagnosis, (**c**) AJCC T stage, (**d**) AJCC N stage, (**e**) AJCC M stage (**f**) AJCC stage, (**g**) radiotherapy, (**h**) chemotherapy and (**i**) treatment. Log-rank test was utilized to compare curves, and significance is presented on each panel

**Fig. 3:**
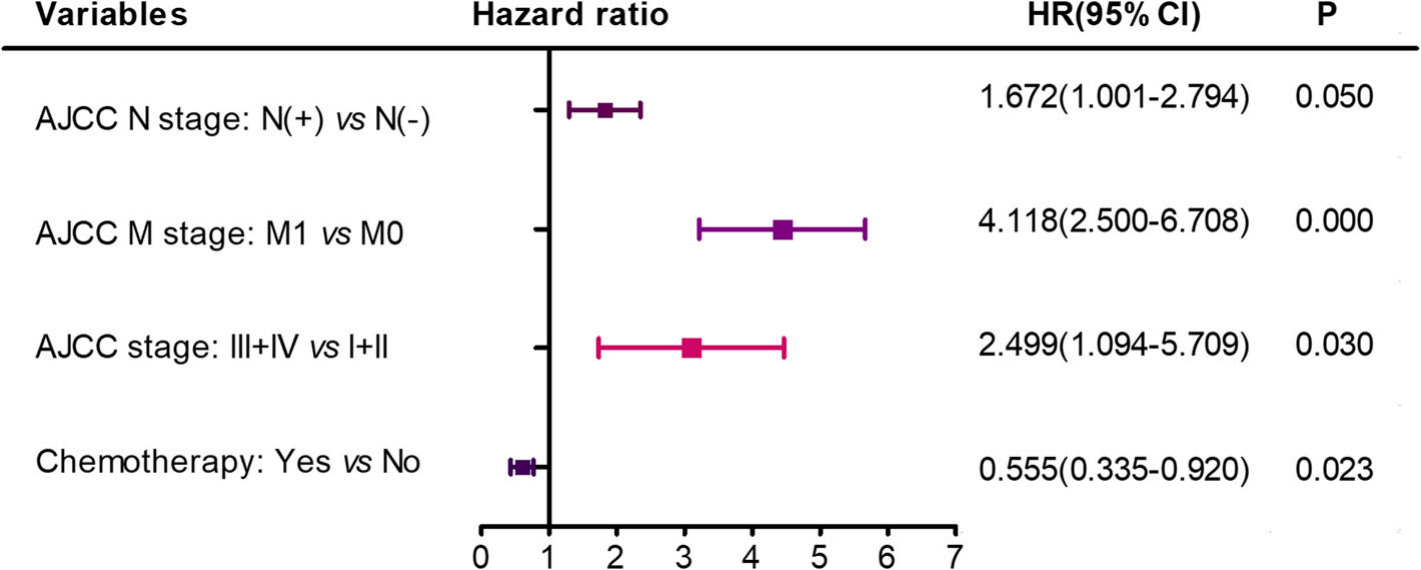
Disease specific independent prognostic factors of H&NSmCC

Cox proportional hazards regression models were built to evaluate the prognostic indicators of overall survival (OS) and DSS via univariate and multivariate survival analyses (Figs. 2 and 3). Patient age, pathological grade, AJCC stage, TNM stage, radiotherapy and chemotherapy significantly affected OS in univariate analysis. In the multivariate analysis, T3 + T4 stage [hazard ratio (HR) 95% confidence interval (CI) = 1.927(1.03–3.61), *P* = 0.04, T1 + T2 as reference (Ref)] and radiotherapy [HR (95% CI) = 2.39(1.41–4.04), *P* = 0.001, without radiotherapy as Ref] were independently associated with OS. Patient age, AJCC stage, N and M stage, radiotherapy and chemotherapy significantly affected DSS in univariate Cox analysis. In the multivariate Cox analysis, AJCC III + IV stage [HR (95% CI) = 2.5(1.1–5.71), *P* = 0.03, I + II stage as Ref], positive N stage [HR (95% CI) = 1.67 (1.01–2.8), *P* = 0.05, negative N stage as Ref], positive M stage [HR (95% CI) = 4.12(2.5–6.71), *P* = 0.000, negative M stage as Ref] and without chemotherapy [HR (95% CI) = 0.56(0.34–0.92), *P* = 0.023, received chemotherapy as Ref] were independently associated with DSS (Fig. 3).

### Prognostic nomogram construction

**Fig. 4:**
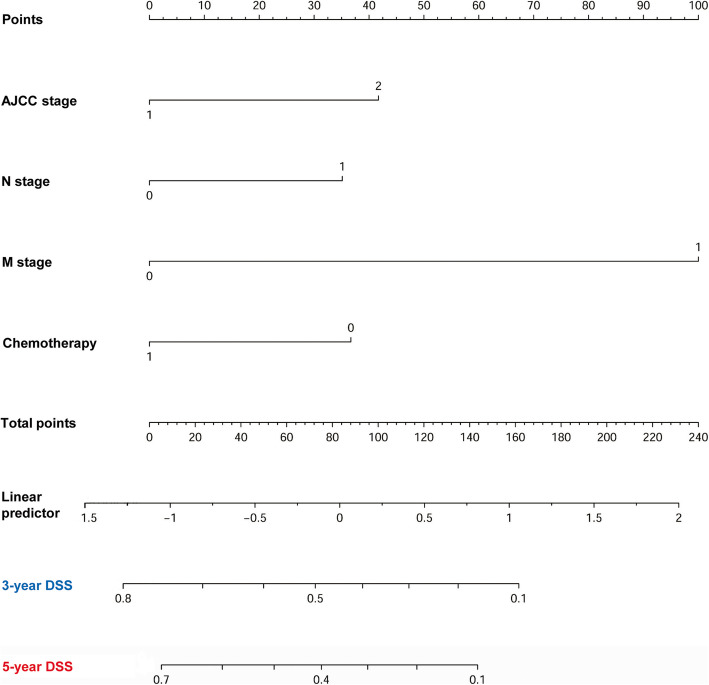
Nomogram for predicting 3- and 5-year disease specific survival found of patients with HNSmCC

**Fig. 5:**
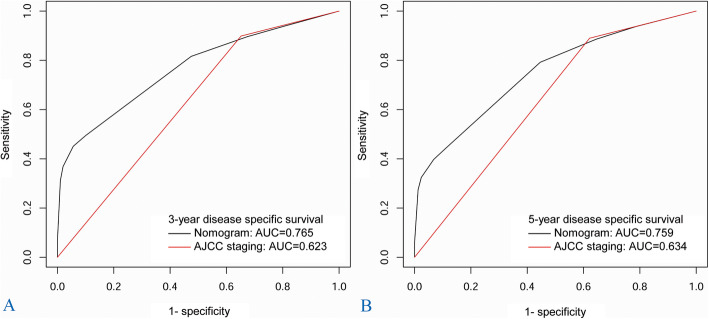
Time-dependent receiver operating characteristics curves of nomogram and AJCC staging system. **a** 3-year disease specific survival and (**b**) 5-year disease specific survival

**Fig. 6:**
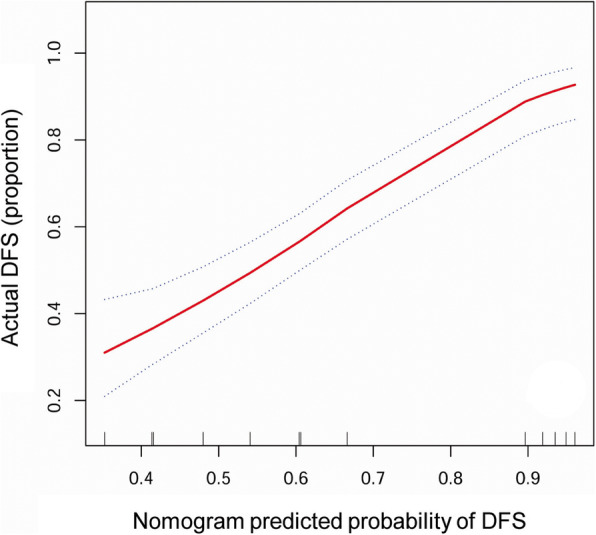
Calibration plots of nomogram

An H&NSmCC-specific nomogram that contained the independent prognostic factors was constructed (Fig. 4). The nomogram demonstrated that the H&NSmCC AJCC M stage had the largest contribution to survival. Each subtype within these variables was assigned a score on the point scale. By adding up the total score and locating it on the total point scale, it could be easily able to draw a straight line down to determine the estimated probability of 3-year DSS and 5-year DSS at each time point. We also compared the predictive ability of the nomogram and the AJCC 7th staging system by calculating the time-dependent AUCs of the ROC curves. The results illustrated that the nomogram could better predict 3- and 5-year DSS (3- year DSS AUC: 0.765 vs 0.623; 5- year DSS AUC: 0.749 vs 0.634). In addition, the C-index of the nomogram (C-index: 0.7041; 95% CI, 0.659 to 0.742) was clearly higher than that of the AJCC 7th staging system (C-index: 0.5873; 95% CI, 0.554 to 0.621) (Figs. 5 and 6).

## Discussion

Our study, based on the SEER database, contains the largest samples in the field of SmCCs in the head and neck region. We described the clinicopathological features of the tumor and assessed the factors that influenced the survival of the cancer. We further established an H&NSmCC-specific nomogram model to predict the survival of H&NSmCC patients based on independent prognostic factors. Because of the infrequent occurrence of the tumor, previous reports discussing H&NSmCC were mainly based on case reports or the experience of a single institution, which were relatively less comprehensive and less objective than our study. The final results of this study are of significance for management and can help doctors estimate the risk of patients with H&NSmCC.

Concerning the clinicopathological characteristics of H&NSmCC, Wakasaki et al. reported that the median age was 74 years (53–91 years) based on 21 cases [11]. Consistent with this result, in this cohort, the median age of all patients was 64 years (2–96 years), and the incidence peaked during the period of 50 to 70 years old. This result suggests that the patients in our study are relatively younger. The data concerning sex also corresponded to the data reported in previous literature. A study reported that most patients with the disease (73.0%) were male [12]. In this study, the male to female ratio was 1.85:1. Our results also showed that white people were more affected by H&NSmCC (86.69%), but there was no significant difference in survival among races. Almost all patients with H&NSmCC in the study had advanced tumor stage, poorly differentiated and undifferentiated tumors totally accounting for 98% of all tumors, which further supports the highly aggressive behaviors of the cancer.

SmCCs of the salivary gland and the nasal cavity had a relatively better prognosis than SmCCs in other regions of the head and neck, while SmCCs of the thyroid gland had the worst survival. Older age, aggressive stage of the tumor, large tumor sizes, lymph node invasion and distant metastasis were correlated with poor prognosis. Consistent with that for other tumors, younger patients had relatively better survival than older patients [13]. Age was an independent prognostic indicator in one study [12]. Tumor grade also influenced the survival of H&NSmCC. Patients with the cancer of poor differentiation or undifferentiation pathological grade had lower survival, however, the results showed no statistically significant difference. Patients with T1 and T2 cancer had longer survival times than the other patients in our study. Similar results have been described in previous reports [12, 14]. These findings demonstrated the importance of tumor size for prognosis, but T stage was not an independent predictor in the nomogram model of H&NSmCC in the current study.

Lymph node metastasis and distant metastasis were identified as independent prognostic indicators in a previous study [5]. Our data further confirmed this conclusion. However, concerning lymph node metastasis, some controversial results have been reported. Walters et al. found that lymph node metastasis was closely related to poor survival, but another report suggested that nodal metastasis had no influence on survival [15, 16]. Our data supported the adverse impact of lymph node metastasis on survival in H&NSmCC. A previous study observed that patients with SmCC of the salivary gland at a distant stage had worse survival than those with limited tumors [15]. Another study containing 344 cases of parotid SmCC showed that distant metastasis was a significant prognosticator in the multivariate model [12]. The locations of distant metastasis were mainly concentrated in the brain, liver, lung and bones [14]. In the present study, 38.28 and 22.94% of patients with H&NSmCC were classified into distant stage and M stage, respectively, and M stage, as another influencing factor, was closely related to prognosis. H&NSmCC is highly sensitive to chemotherapy, however, due to extremely aggressive behaviors of the cancer, tumor cells cannot be eradicated at M1 stage and H&NSmCC is likely to relapse after chemotherapy and radiotherapy, leading to a worse prognosis for patients with distant metastases [1, 7].

Radiotherapy and chemotherapy are considered the mainstays of treatment [1]. Surgery has only a very limited role in locoregional lesions [1]. In the limited stage, surgical resection is suggested and can improve survival [17]. Radiotherapy and chemotherapy, as adjuvant treatment, can largely extend patients’ life expectancy [8, 18]. Radiotherapy is effective in palliating intrathoracic symptoms, such as chest pain, shortness of breath and other systemic symptoms due to the metastasis of tumors. Platinum-based chemotherapy regimens, the most frequent therapy, lead to better survival than earlier chemotherapy [18]. Cisplatin/etoposide was also reported to have a high response for H&NSmCC [19]. Prophylactic cranial irradiation (PCI) is still controversial as a routine therapy for H&NSmCC [20, 21]. In the small lung carcinoma, the recommended dose of PCI is 25 Gy in 2.5-Gy fractions or 30 Gy in 1.8-Gy to 2-Gy fractions for H&NSmCC patients [22, 23].

Patients receiving chemotherapy, radiotherapy and surgery had the longest survival in our study, but the comparison of the three cohorts of treatments showed no significant differences. In addition, owing to the diversity of the clinicopathological features among patients, the results could not demonstrate that the combination of surgery, chemotherapy and radiotherapy was the most appropriate therapy for all H&NSmCC patients. Both radiotherapy and chemotherapy were vital for the survival of H&NSmCC patients in our study, but only chemotherapy was an independent prognostic factor in the predictive model, which highlighted the importance of chemotherapy for the tumor. Interestingly, patients receiving chemotherapy had a worse survival than others, which might be because patients with chemotherapy mostly had advanced stage tumors, and thus, the survival was not satisfactory. Due to the low incidence, standard treatment is still to be confirmed in the future.

In the current study, we constructed a specific prognostic model for H&NSmCC patients. The model can better predict the prognosis of H&NSmCC than the AJCC staging system as the information from the SEER database, such as age, pathological grade, AJCC stage and treatment, were all considered and therefore the result of the model is closer to the actual prognosis. Of course, there are some limitations of the current investigation. First, lack of detailed comorbidity data represents one of biggest limitation of SEER database. Second, the study has shortcomings due to its retrospective nature. Third, information on H&NSmCC patients was not complete, so we failed to analyze several factors that may influence the prognosis of the cancer. For instance, patients with information on surgery and T stage only accounted for 42.3 and 45.1%, respectively. Only one patient did not receive surgery which is not adequate for survival analysis. Owing to the limited number of patients with clear T stage, the factor was not included in the nomogram model. Fourth, the nomogram should be applied to patients with caution. Because it was built based on the retrospective investigation with lower level of evidence.

## Conclusion

In summary, our study analyzed the clinicopathological features and treatment outcomes of H&NSmCC based on the SEER database, one of the largest cancer databases in the world. H&NSmCC, with very low occurrence, had a poor prognosis. The incidence peaked during the period of 50 to 70 years old, and the most frequent location in the cohort was the salivary gland. The 5-year OS and 5-year DSS were 26.2 and 27%, respectively, for H&NSmCC. N stage, M stage AJCC stage and chemotherapy were independent prognostic indicators. The nomogram model was constructed according to the above indicators. The model can better predict the survival of H&NSmCC patients than the AJCC staging system and facilitates doctors in the assessment of patient prognosis and helps patients choose reasonable treatments.

## Data Availability

Study data was publicly available in the SEER database.

## Abbreviations

SmCCs: Small cell carcinomas
H&NSmCC: Head and neck SmCCs
SEER: Surveillance, Epidemiology and End Results
ICD-O-3: International Classification of Diseases in Oncology, third edition
AJCC: American Joint Committee on Cancer
C-index: Concordance index
AUC: Area under the curve
ROC: Receiver operating characteristic
SPSS: Statistical Package for Social Sciences
DSS: Disease-specific survival
OS: Overall survival
HR: Hazard ratio
CI: Confidence interval
Ref: Reference
